# The Prevalence of Tonsilloliths and Other Soft Tissue Calcifications in Patients Attending Oral and Maxillofacial Radiology Clinic of the University of Iowa

**DOI:** 10.1155/2014/839635

**Published:** 2014-01-22

**Authors:** Babatunde Olamide Bamgbose, Axel Ruprecht, John Hellstein, Sherry Timmons, Fang Qian

**Affiliations:** ^1^Department of Oral and Diagnostic Sciences, Faculty of Dentistry, Bayero University, Kano, Nigeria; ^2^Department of Oral Pathology, Radiology, and Medicine, College of Dentistry and Dental Clinics, The University of IA, Iowa City, IA, USA; ^3^Department of Preventive and Community Dentistry, College of Dentistry and Dental Clinics, The University of Iowa, Iowa City, IA, USA

## Abstract

*Objective*. The purpose of this study was to determine the prevalence of tonsiliths in patients attending the oral and maxillofacial radiology clinic of The University of Iowa and to determine if there is any correlation between the presence of tonsiliths and the presence of stones in other body tissues, ducts, or organs. *Study Design*. This was a two-part study. The first part was a prevalence study whereas the second was a matched pair case-control study. The matched pair case-control study commenced after the prevalence study was concluded. No new or unusual radiographs were made in this study. The study only reviewed radiographs that were made for clinical purposes. *Results*. A total of 1524 pantomographs were reviewed and 124 subjects (53 males and 71 females) aged 9 years and 2 months to 87 years (mean age 52.6 years) were included for data analysis. Thirty-eight subjects had single tonsiliths whereas 86 subjects had multiple tonsiliths. The prevalence of tonsiliths in the study population was 8.14%. A total of 20 subjects were included in the second part of the study, comprising 10 each for matched pair case-control groups. The observations did not indicate any correlation between the presence of tonsiliths and the presence of stones in other body tissues, ducts, or organs. *Conclusion*. The prevalence of tonsiliths in our study population was 8.14%. The observations in our study do not support any correlations between tonsiliths and calcifications in other body tissues, organs, or ducts.

## 1. Introduction

### 1.1. Tonsiliths

Tonsiliths, also known as tonsilloliths and tonsillar concretions or simply called liths, are stones that arise from calcium being deposited on desquamated cells and bacterial growth in the tonsillar or adenoidal crypts and occur in patients with or without a history of inflammatory disorders of either the tonsils or adenoids [1, 2]. Tonsiliths may be associated with symptoms, including nonspecific chronic sore throat, irritable cough, dysphagia, otalgia, chronic halitosis, a foreign body-like sensation, or foul taste [1–7]. Patients with tonsiliths may also be asymptomatic, with the liths discovered incidentally on pantomographic or lateral cephalometric radiographs [7–9]. Superimposition of hard and soft tissue structures on such radiographic images is common, creating a diagnostic challenge. This necessitates the consideration of several interpretations of radiopacity in the mandibular molar-ramus region including sialolith, tonsilith, phlebolith, calcified lymph node, carotid artery arteriosclerosis, stylohyoid ligament ossification, and dystrophic calcification in acne scars [3]. These entities can be differentiated by the radiographic features and locations.

**Figure 1:**
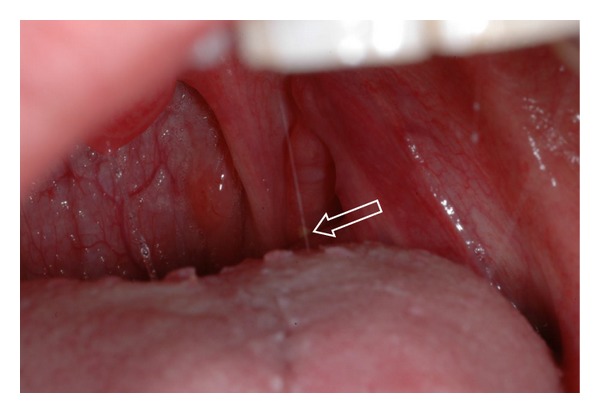
Clinical photograph of a patient with small, superficial tonsilith in the left tonsillar crypt (arrow).

On clinical examination, a superficial tonsilith may be seen as a white or yellowish hard mass within the tonsillar crypt [10]. The tonsilith may also have a deeper location and present as an enlarged or calcified mass within the tonsil [10]. Superficial tonsiliths often flake off periodically, especially when the patient gargles vigorously (Figure 1). Tonsiliths can be multiple and may vary in size from small to very large [4–10], the largest tonsilith to date, measuring 14.5 cm, was reported by Rubin [11] in 1936.

Treatment is usually removal of the tonsilith by curettage. Larger concretions may require local excision under topical or local infiltration anesthesia. If there is evidence of chronic tonsillitis, tonsillectomy offers definitive therapy [5, 10].

### 1.2. Composition of Tonsiliths

Tonsiliths are composed of phosphate and/or carbonate salts of calcium. These are arranged in a structure similar to that of bone crystals of hydroxyapatite Ca_5_[OH | (PO_4_)_3_]. The hydroxyl ion (OH^−^) in the hydroxyapatite can be replaced by fluoride, carbonate, or chloride. The hydroxyapatite crystal has a specific gravity of 3.08 and is 5 on the Mohs hardness scale [10, 12]. A protein matrix has also been demonstrated as part of the composition of tonsilith [12–14].

### 1.3. Radiology of Tonsiliths

**Figure 2:**
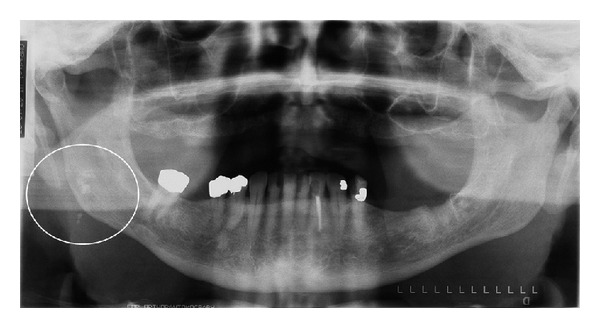
Multiple, well-defined calcifications in the right tonsil (circle) in a 79-year-old male.

**Figure 3:**
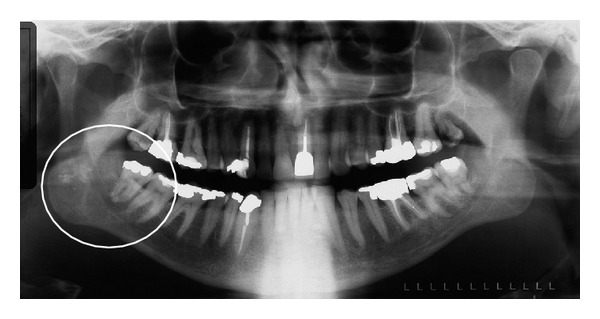
Multiple, well-defined calcifications in the right tonsil (circle) in a 54-year-old male.

**Figure 4:**
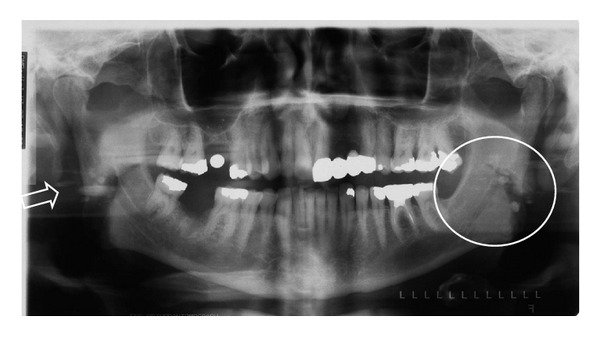
Multiple, well-defined, bilateral calcifications in the angle-ramus region of the mandible (circle) in a 57-year-old male. Note ghost images on the right (arrow).

**Figure 5:**
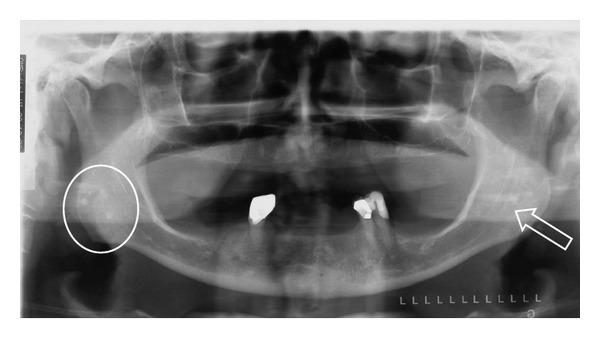
Multiple, well-defined, and bilateral calcifications in the angle-ramus region of the mandible (circle and arrow) in a 67-year-old female.

Although a pantomograph is a reliable and standard modality for interpreting the presence of tonsiliths, superimposition of a lesion involving one side of the jaw may create a pseudotonsilith or ghost image on the contralateral side which could lead to a misinterpretation of bilateral lesions [15]. A ghost image is formed when the object is located between the X-ray source and the center of rotation of the cassette [16–19]. On the pantomograph, tonsiliths commonly appear as multiple, small, and ill-defined radiopacities, (Figures 2, 3, 4, and 5).

*Other Stones*. Stones or liths can occur in various organs and ducts in the body, including the gallbladder (cholelith), kidneys (nephrolith), and lower urinary tract (urolith). Liths are also seen in the nasolacrimal duct (dacryolith), nasal cavity (rhinolith), maxillary antrum (antrolith), lymph nodes, liver (intrahepatic lith), testes (testicular microlith), intestine (fecalith), and the semicircular canal (canalolith) [17, 20–22]. Other liths include dental calculus, ocularlith, pancreatolith, sialolith, and phlebolith.

## 2. Materials and Methods

Approval for both parts of the study was granted by the University of Iowa Institutional Review Board. This study complied with the Helsinki Declaration as regards human subjects. This was a two-part study. The first part was a prevalence study while the second was a matched pair case-control study. The matched pair case-control study commenced after the prevalence study was concluded. No new or unusual radiographs were made in this study. The study only reviewed radiographs that were made for clinical purposes.

### 2.1. Part 1: Prevalence Study

The intended population for the prevalence study comprised all the patients who attended the oral and maxillofacial radiology clinic of the College of Dentistry of The University of Iowa over a four-year period, between January 2000 and December 2003. The study population included both subjects with and without tonsiliths found on pantomographs. The age range was three to ninety years. A total of 1524 subjects were recruited.

Only subjects with diagnostic quality pantomographs were included because these radiographs could potentially show calcifications in the tonsils over the angle and ramus of the mandible.

In order to determine prevalence, subjects with and without tonsiliths demonstrable on the pantomographs were included in the study. Both analog and digital pantomographs were included.

Individuals below the age of three or whose pantomographs were of poor diagnostic quality or with a history of tonsillectomy were excluded.

Tonsiliths measuring 2 mm or less were excluded because this dimension did not give a high confidence level of interpretation.

The radiographs were viewed in the interpretation room in subdued lighting over a clean and bright illuminator or computer screen. A magnifying glass or digital magnification was used to assist in viewing the concretions on the radiographs.

#### 2.1.1. Ascertainment of Tonsiliths

An interpretation of tonsiliths was made when radiopaque masses not deemed to be part of the stylohyoid complex, sialoliths, calcified lymph nodes, phleboliths, or changes in the bone pattern were seen over or near the angle and ramus of the mandible. The concretions needed to have a radiopacity similar to, but demarcated from, the overlying bone. Unilateral and bilateral concretions were recorded. Ghost images were identified to prevent errors of interpretation. The size, site, and number of concretions were recorded. Unique identifiers (chart numbers) of the subjects were recorded.

The principal investigator was calibrated against multiple observers such that a capital value of agreement was established. This was done in order to establish criteria for interpreting tonsiliths. The exercise was repeated halfway through the study to prevent a drift from established standards.

A standardized chart abstraction form was used to extract relevant information from the dental records of the subjects. The information extracted included demographics such as age and sex and radiographic appearance of tonsiliths.

The lack of previous data or studies made it difficult to estimate the sample size. Therefore, a convenience sample inclusive of all the subjects who attended the oral and maxillofacial radiology clinic from January 2000 to December 2003 was used.

#### 2.1.2. Statistical Analysis

Descriptive statistics were computed, and frequency tables were generated. Bivariate analyses were performed to determine if there was a statistically significant difference between two groups of patients (with single tonsiliths versus with multiple tonsiliths) for demographic factors. Chi-square test, Fisher's exact test, and nonparametric Wilcoxon rank-sum test were conducted for the data analysis.

All tests had a 0.05 level of statistical significance. SAS for Windows (version 9.1, SAS Institute Inc., Cary, NC, USA) was used for the data analysis.

### 2.2. Part 2: Matched Pair Case-Control

The second part, the matched pair case-control study, included the rolling enrolment of subjects referred to the department over a six-month period (October 2007 to March 2008). The control group comprised subjects without the radiographic evidence of tonsiliths on pantomographs, whose medical history may or may not reveal presence of calcifications or stones in other tissues and organs, including kidneys and salivary glands. The case group comprised subjects with radiographic evidence of tonsiliths on pantomographs, who may or may not have history of calcifications or stones in other tissues or organs, including the kidneys and salivary glands. For each subject with tonsiliths, that was enrolled into the case group, another potential subject without tonsiliths was recruited to the control group. The subjects recruited to the control group were matched for age (±2 years) and sex with the subjects in the case group. The subjects for both case and control groups were enrolled from the patients attending the oral and maxillofacial radiology clinic.

The involvement of subjects consisted of one clinical visit of approximately 30 minutes. No additional clinic follow-ups were necessary. There were no foreseeable risks from participating in the study.

#### 2.2.1. Selection Criteria

*Inclusion Criteria*

1. Age range: 3–90 years.
2. Only subjects without tonsiliths on pantomographs were included in the control group.
3. Only subjects with tonsiliths on pantomographs were included in the case group.
4. Diagnostic quality pantomograph.

The features of a diagnostic quality pantomograph include

- both rami of the mandible visible on the radiograph;
- proper positioning of the patient in the unit when the radiograph was made;
- areas of the tonsils included in the image;
- the overall density of the radiograph acceptable for interpretation;
- no artifacts over the rami of the mandible to preclude the visualization of tonsiliths.

*Exclusion Criteria*

1. Subjects below the age of three years.
2. Subjects with poor diagnostic quality pantomographs.
3. Subjects who declined to give informed consent.
4. Subjects with a history of tonsillectomy.

#### 2.2.2. Statistical Analysis

Descriptive statistics were computed and frequency tables were generated. Bivariate analyses were performed to determine whether there was a statistically significant difference between the case and control groups. Chi-square test, Fisher's exact test, and nonparametric Wilcoxon rank-sum test were used for data analysis.

All tests have a 0.05 level of statistical significance. SAS for Windows (version 9.1, SAS Institute Inc., Cary, NC, USA) was used for the statistical analysis.

To our knowledge, no similar studies have been conducted before and, therefore, no analogous information is currently available for determination of sample size of case-control groups. This initial study, which was completed in approximately 6 months, therefore included all the subjects with a matched pair for case-control study.

The results of the prevalence study that was earlier conducted could not be used to calculate the sample size for the matched, pair case-control study because the data did not include correlations between tonsiliths and other stones in body organs, tissues, and ducts.

## 3. Results

### 3.1. Part 1: Prevalence Study

#### 3.1.1. Descriptive Statistical Results

A total of 1524 pantomographs were reviewed in the oral and maxillofacial radiology clinic of The University of Iowa. 205 subjects with possible tonsiliths were identified. Of the 205 subjects, 124 had tonsiliths over 2 mm in size with a high confidence level. The 81 subjects eliminated possibly had tonsiliths, but the 2 mm dimension did not give a high confidence level of interpretation.

Therefore, 124 subjects (53 males and 71 females) aged 9.2 to 87 years (mean age 52.6 years) were included for the data analysis. The male-to-female ratio was 0.75 : 1.00. Thirty eight subjects had single tonsiliths whereas 86 subjects had multiple tonsiliths. The prevalence of tonsiliths in the study population was 8.14%.

Table 1 displays the frequency distribution analyses for some categorical variables, and Table 2 reports the descriptive statistics for some quantitative variables.

#### 3.1.2. Comparison of Two Groups of Subjects (with Single Tonsilith versus Multiple Tonsiliths)

Based on chi-square test or Fisher's exact test, the data revealed that there was a significant difference between the two groups for the unilateral right side of the jaw (*P* = 0.0242) and bilateral side of the jaw (*P* < 0.0001). The results indicated that single tonsiliths were more likely to be on the right side of the jaw (55.3%). On the other hand, multiple tonsiliths were more likely to present on both sides of the jaws (37.2%).

Based on the nonparametric Wilcoxon rank-sum test, the data showed that there was no significant difference in mean age and mean size of tonsiliths between the two groups of subjects (with single tonsilith versus multiple tonsiliths) (*P* = 0.4318 and 0.0551, resp.).

Based on chi-square test or Fisher's exact test, the data revealed that there was no significant difference between the two groups for sex (*P* = 0.6248), location over the ramus of the mandible (*P* = 0.6998), unilateral left presentation (*P* = 0.1549), and presence of ghost images (*P* = 0.6998).

#### 3.1.3. Comparisons of Locations for Tonsiliths

No significant results were found (*P* > 0.05 for all instances).

### 3.2. Results of Matched Pair Case-Control Study on the Prevalence of Tonsiliths

#### 3.2.1. Descriptive Statistical Results

A total of 20 subjects were included in this study, comprising 10 each for the matched pair case and control groups. The age range was 17–83 years, with a mean age of 42.95 years and standard deviation of 22.09. The sex ratio was 4 : 1 for both the case and the control groups.

A total of 8 subjects (40%) provided a history of recent episodes of tonsillitis. But 2 out of the 8 subjects (10%) did not provide a record of the date of the last episode of tonsillitis. Out of the 6 subjects with a record of the last date of tonsillitis, 4 (67%) were in the case group while 2 (33%) were in the control group (Table 3). The number of episodes of tonsillitis per year and the side(s) affected are depicted in Tables 4 and 5.

The subjects gave varied responses to questions about the symptoms of tonsiliths. The symptoms included the history of chronic sore throat, chronic cough, difficulty in swallowing, pain in the ear, chronic halitosis, foreign body-like sensation in the throat, and bad/altered taste. The observations are presented in Table 6.

Two subjects gave a history of tonsillectomy for the management of tonsillitis. These subjects were excluded from the data. Two other subjects (10%) gave a history of antibiotic regimen for the management of tonsillitis. There was no record of curettage in the observations.

The medical history of stones in other body tissues, organs, and ducts was sought by asking questions about sialoliths, phleboliths, arteriosclerosis, calcified lymph nodes, dacryoliths, antroliths, rhinoliths, nasopharyngeal stones, nephroliths, and gall bladder stones. One subject each had a history of phlebolith and calcified lymph nodes, respectively. There was no other history of body tissue concretions.

A total of 5 (25%) gave a history of cigarette smoking. Out of the sample, only three still smoke regularly. The date of last dental cleaning at a dental facility and calculus distribution on the lingual and facial surfaces of the mandibular incisors and maxillary molars were recorded for each subject. The date of last professional cleaning from a dentist ranged from less than 1 week to 10 years. The observations are presented in Table 7.

A total of 13 (65%) pantomographs were analog, whereas 7 (35%) were digital. A total of 18 (90%) pantomographs were made on the OC 100-3-1-2 machine, whereas the remaining 2 (10%) were made on the OP 100-3-1-2 machine.

The radiographic appearance of tonsiliths was categorized as single, well-defined (*n* = 3, 15%) and multiple, well defined (*n* = 7, 35%). The location of tonsiliths, side of the jaw, and ghost images were also recorded. The observations are presented in Table 8.

The tonsils were examined clinically. The observations showed that all the tonsiliths (*n* = 10, 50%) were located deep in the tonsillar crypt and were not visible to the naked eye.

The largest tonsilith in the radiographic observation was 6 mm, whereas the smallest was 3 mm (Table 8).

The mean values of observations for the case and control groups are presented separately on Tables 9 and 10.

#### 3.2.2. Statistical Results on Comparison of Case Group versus Control Group for Selected Variables in the Study

Based on the nonparametric Wilcoxon rank-sum test, the data revealed that there was a significant difference between case and control as the date of a last professional cleaning was received from a dental facility (*P* = 0.0105). The results indicated that the average weeks for last professional cleaning at the dental facility for subjects in the case group (mean = 226.88 weeks) were significantly greater than those observed in the control group (mean = 43.5 weeks).

Based on Fisher's exact test, the data revealed that there was no significant difference between case and control groups for tonsillitis, side(s) affected, sore throat, chronic cough, difficulty swallowing, pain in ear, chronic halitosis, foreign body feel, antibiotics, phlebolith, calcified lymph node, use of tobacco products, and dental calculus (*P* > 0.05 for each instance).

Based on the nonparametric Wilcoxon rank-sum test, there was no significant difference between case and control groups for the date of last episode of tonsillitis (*P* = 0.1588), number of episodes of tonsillitis per year (*P* = 0.8308), when the subject quit tobacco use (*P* = 0.3711), average number of cigarettes smoked per day (*P* = 0.7671), and age distribution of patients (*P* = 0.9396).

## 4. Discussion and Conclusion

This study was designed as a two-part study to determine the prevalence of tonsiliths and the relationship between tonsiliths and concretions in other body organs, tissues, or ducts in the patients attending the oral and maxillofacial radiology clinic of The University of Iowa. In the first part of the study, a total of 1524 charts, representing pantomographs made between January 2000 and December 2003, were reviewed. One hundred and twenty-four (8.14%) cases of tonsiliths, measuring above 2 mm on a linear scale were included in the final analysis. Eighty-one cases with possible tonsiliths, measuring 2 mm and below, were removed from the data because this dimension does not give a high confidence level of interpretation. Therefore, the prevalence of tonsiliths in patients attending the oral and maxillofacial radiology clinic of The University of Iowa was observed to be 8.14%. This figure is similar to what was reported by Cooper and his coworkers in 1983 [12]. The minimum size of tonsilith in the Cooper study was 5 mm. An important aspect of our study is that we established a cut-off point of 2 mm for the interpretation of tonsiliths based on our reliability of interpretation and high degree of consensus of this measurement amongst the researchers in this study. This is not a common feature in all the previous studies reviewed.

The earliest known description of concretions in the oropharynx is thought to be recorded by Lang in 1560 [12, 23]. Although many views have been expressed, the exact etiology and pathogenesis are unknown [24]. It has been shown that the calcifications develop within a mass of desquamated epithelium, serum, food debris, and bacterial colonies. Recurrent tonsillar inflammation may promote the development of tonsillar concretions [25].

Out of the 124 cases of tonsiliths in our study, 53 were males and 71 were females. The male-to-female ratio was 0.75 : 1.00 and there is no sex predilection. The age range of subjects was 9.2–87 years (mean 52.6 years). The average size of tonsilith was 4 mm (range: 3–11 mm). The incidence of large tonsiliths is low. They are seen mainly in elderly patients [24, 26–28]. On the other hand, small tonsiliths are frequently reported [1, 4, 7, 10–12, 18, 24, 26–28]. A few of the subjects in our study mentioned that they often see fragments of calcific materials in their sputum, especially during episodes of tonsillitis. This may account for the low prevalence of large tonsiliths, especially in healthy individuals. According to the literature review by Cooper and his co-workers [12], the average age of patients at the time of medical intervention is 46.4 years (41.6 years for males, 50.7 years for females). A review of the English literature showed that the mean age of occurrence was 46.2 years (age range: 16–77 years) [1, 4, 7, 10–12, 18, 20, 24, 26–28].

The radiographic appearance of tonsiliths in this study was predominantly multiple and well defined (*N* = 78, 62.90%). The single, well-defined tonsilith in a similar location constituted 28.23% (*N* = 35) of the sample. Tonsiliths are usually single and unilateral, although they can be multiple and bilateral [26]. They may grow to large sizes and may occur in palatine or lingual tonsils [24].

The majority of the cases were located in the lower one-third of the mandibular ramus (*N* = 116, 93.55%). This is not unusual, inasmuch as the tonsils are found in the lateral aspect of the oro pharyngeal wall corresponding to the angle of the mandible bilaterally [14, 15]. There was a higher percentage of tonsiliths (*N* = 50, 40.32%) in the right tonsillar crypt than in the left (*N* = 41, 33.06%). A similar distribution was observed in the smaller sample for the matched pair case-control study. It is also not uncommon to have bilateral tonsiliths (*N* = 33, 26.61%). According to the literature review by Ram and his co-workers [15], tonsiliths occur more often in the right tonsil than in the left one.

The mean age of subjects with multiple and well-defined concretions was slightly higher (53.68 years) when compared to the mean age of subjects with single, well-defined concretions (50.32 years). A similar pattern was observed with the average size of tonsiliths, which is 4.18 mm for single concretions and 3.78 mm for multiple concretions.

For the matched pair case-control study, the age range of subjects was 17–83 years (*N* = 20). The male-to-female ratio of 4 : 1 may be attributable to the small sample size, although Ram et al. [15] in their literature review concluded that tonsiliths occur twice as commonly in males than females. It was observed that tonsillitis is a fairly common finding in cases with tonsiliths (*N* = 8, 40%). The tonsillitis appears to be chronic and bilateral with at least one episode per year (range: 1–6). Chronic cough (*N* = 2), chronic halitosis (*N* = 1), difficulty in swallowing (*N* = 1), and a foreign body-like sensation were some of the other symptoms associated with tonsiliths in our study population.

The observations in this study do not indicate any statistical difference in tobacco use between the case and control groups. It is not clear if any association exists between tobacco use and tonsiliths.

Patients with tonsiliths may be asymptomatic and their tonsiliths may be discovered incidentally on pantomographs or other imaging modalities, including CTs and MRIs [23]. Symptomatic patients may present with a wide range of symptoms and signs, including pain, dysphagia, enlarged tender neck glands, a lump in the throat, halitosis, and ear pain. Clinical examination reveals a white or yellowish hard object within the tonsillar crypt [23].

There has been growing interest in the association between tonsiliths and halitosis [21, 29–31]. Tsuneishi and co-workers [30] reported the composition of the bacterial flora of tonsiliths in their analysis of six subjects with tonsiliths. Their study showed that tonsiliths have both aerobic and anaerobic bacteria. Some of the anaerobic bacteria produce volatile sulfur compounds such as hydrogen sulfide and methyl mercaptan. The volatile sulfur has been implicated in halitosis [29, 31].

The observations in our study do not support any correlations between tonsiliths and calcifications in other body organs, tissues, or ducts. One subject gave a history of calcified lymph nodes and phlebolith. The study population did not indicate any history of sialoliths, dacryoliths, antroliths, nephroliths, or gall bladder stones. When correlated with calculus in the oral cavity, 11 of the subjects (55%) had calculus on the surfaces of the mandibular incisors and maxillary molars. The facial surfaces of the maxillary molars and the lingual surfaces of the mandibular incisors were selected because the high concentration of calcium at the opening of the Stenson and Warthon's ducts translates to higher concentration of calculus at the locations. Saliva is supersaturated with calcium and phosphate and calculus derives its calcium from saliva. Inasmuch as saliva also percolates the tonsils and tonsillar crypts, it is assumed that the calcium and phosphate content of saliva may play a role in the formation of tonsiliths. Furthermore, the salivary calcium regulatory protein, statherin, may also have a role to play in the formation of tonsiliths. It is, therefore, possible that there is a correlation between salivary calcium and tonsiliths.

*Limitations of Study*. The small sample size for the matched pair case-control study made it difficult to do extensive statistical analysis of the variables. Also, six months did not appear adequate to fully explore all the variables in this research study.

Furthermore, the responses to the history of possible calcifications in other body tissues, ducts, or organs were based solely on study subject recall of past medical history. This information was rather subjective and a better assessment may have been obtained if the history provided by the study subject was compared to medical records.

## Figures and Tables

**Table 1:** Frequency distribution of sex, imaging modalities, radiographic appearance, location, side of jaw, ghost images, and size of tonsiliths.

	Frequency (*N*)	Percent (%)
Sex		
Male	53	42.74
Female	71	57.26
Total	**124**	**100.00**
Imaging modalities		
Plain film	124	100.00
Digital images	None	0.00
Total	**124**	**100.00**
Radiographic appearance of tonsiliths		
Single, welldefined	35	28.23
Single, illdefined	3	2.42
Multiple, welldefined	78	62.90
Multiple, ill-defined	8	6.45
Total	**124**	**100.00**
Location of tonsiliths		
Upper 1/3 of mandibular ramus	0	0.00
Middle 1/3 of mandibular ramus	8	6.45
Lower 1/3 of mandibular ramus	116	93.55
Total	**124**	**100.00**
Side of jaw		
Unilateral left	41	33.06
Unilateral right	50	40.32
Bilateral	33	26.62
Total	**124**	**100.00**
Ghost images		
Present	8	6.45
Not present	116	93.55
Total	**124**	**100.00**
Size of tonsiliths (mm)		
3 < 4	69	55.65
4 < 6	24	19.35
6 < 7	17	13.71
7 < 8	10	8.06
8 < 9	1	0.81
9 < 10	1	0.81
10 < 11	1	0.81
11	1	0.81
Total	**124**	**100.00**

**Table 2:** Mean of age of subjects and size of tonsiliths for both single and multiple tonsiliths.

Variable	Mean	Standard deviation	Minimum	Maximum
	Subjects with both single and multiple tonsiliths (*N* = 124)			
Age (year)	52.65	20.64	9.20	87.00
Size of tonsiliths (mm)	3.90	1.38	3.00	11.00

	Subjects with single tonsiliths (*N* = 38)			
Age (year)	50.32	21.67	9.20	84.00
Size of tonsiliths (mm)	4.18	1.54	3.00	11.00

	Subjects with multiple tonsiliths (*N* = 86)			
Age (year)	53.68	20.21	13.20	87.00
Size of tonsiliths (mm)	3.78	1.29	3.00	10.00

**Table 3:** Frequency distribution of history of tonsilitis for the matched pair case-control study.

Variable	Frequency (*N*)	Percent (%)
	Subjects in case group (*N* = 10)	
Previous history of tonsilitis	6	60
No previous history of tonsillitis	4	40

	Subjects in control group (*N* = 10)	
Previous history of tonsilitis	2	20
No previous history of tonsillitis	8	80

**Table 4:** Frequency distribution of unilateral and bilateral tonsilitis for the matched pair case-control study.

Side affected	Frequency (*N*)	Percent (%)
	Subjects in case group (*N* = 10)	
Unilateral	1	10
Bilateral	5	50
Not applicable	4	40

	Subjects in control group (*N* = 10)	
Unilateral	None	0
Bilateral	2	20
Not applicable	8	80

**Table 5:** Frequency distribution of the number of episodes of tonsilitis per year for the matched pair case-control study.

Number of episodes of tonsillitis per year	Frequency (*N*)	Percent (%)
	Subjects in case group (*N* = 10)	
1	4	40
2	1	10
6	1	10
Not applicable	4	40

	Subjects in control group (*N* = 10)	
1	2	20
Not applicable	8	80

**Table 6:** Frequency distribution of symptoms of tonsilitis (*N* = 20).

Variable	Frequency	
	Case (*N* = 10)	Control (*N* = 10)
Sore throat	2	0
Chronic cough	2	0
Difficulty swallowing	1	0
Pain in ear	1	0
Chronic halitosis	1	0
Foreign body-like feel	2	0
Altered taste	None	None
Previous history of tonsilith	None	None

**Table 7:** Frequency distribution of dental calculus.

Calculus	Case (*N* = 10)	Control (*N* = 10)
Lingual surfaces of mandibular incisors	1	1
Facial surfaces of maxillary molars	0	2
Lingual surfaces of the mandibular incisors and facial surfaces of the maxillary molars	5	2
No calculus	4	5

**Table 8:** Frequency of radiographic appearance, location, side of jaw, ghost images, and size of tonsiliths.

	Case (*N* = 10)	Control (*N* = 10)
Radiographic appearance		
Single, welldefined	3	0
Multiple, welldefined	7	0
Nil tonsiliths (control group)	0	10
Location of tonsilith(s)		
Lower third of mandibular ramus	10	0
Nil tonsiliths (control group)	0	10
Side of the jaw with tonsilith		
Unilateral left	1	0
Unilateral right	6	0
Bilateral	3	0
Nil tonsiliths (control group)	0	10
Ghost images		
Present	2	0
Not present	8	0
Nil tonsiliths (control group)	0	10
Size of tonsilith seen on radiographs (mm)		
3	2	0
4	3	0
5	3	0
6	2	0
Nil (control group)	0	10

**Table 9:** Mean values of observations for the case group.

Variable	Subjects (*N*)	Mean	Standard deviation	Minimum	Maximum	Median
Last tonsillitis (weeks ago)	4	3.75	3.10	1.00	8.00	3
Episodes of tonsillitis per year	5	2.00	2.35	0.00	6.00	1.00
When quit smoking (weeks ago)	3	312.00	187.49	156.00	520.00	260.00
Num. of cigarettes per day	3	7.33	10.97	1.00	20.00	1.00
Last dental cleaning (weeks ago)	8	226.88	162.92	43.00	520.00	234.00
Size of tonsiliths (mm)	10	4.50	1.08	3.00	6.00	4.50
Age of subject (years)	10	42.70	22.39	17.00	82.00	39.00

**Table 10:** Mean values of observations for the control group.

Variables	Subjects (*N*)	Mean	Standard deviation	Minimum	Maximum	Median
Last tonsillitis (weeks)	2	30.00	31.11	8.00	52.00	30.00
Episodes of tonsillitis per year	2	1.00	0.00	1.00	1.00	1.00
When quit smoking (weeks)	1	104.00	0.00	104.00	104.00	104.00
Number of cigarettes per day	2	6	1.41	5.00	7.00	6.00
Last clean (weeks)	10	43.50	46.22	2.00	156.00	52.00
Size of tonsiliths (mm)	0	0.00	0.00	0.00	0.00	0.00
Age of subjects (yr)	10	43.20	22.99	17.00	83.00	39.00

## Statement of Clinical Relevance

This study was done to, first, determine the prevalence of tonsiliths in patients attending the University of Iowa Dental Clinic and, secondly, to investigate a correlation between tonsiliths and other concretions. Tonsiliths are a major source of halitosis that is often overlooked by clinicians.
